# Bilateral highly differentiated follicular carcinoma of ovarian origin with extensive abdominal metastasis: A case report

**DOI:** 10.1097/MD.0000000000049886

**Published:** 2026-07-17

**Authors:** Lina Wang, Hao Dong, Cuncheng Lou, Qiong Fu, Yonggang Qiu

**Affiliations:** a Department of Radiology, The First People’s Hospital of Xiaoshan District, Xiaoshan Affiliated Hospital of Wenzhou Medical University, Hangzhou, Zhejiang, China; b Department of Pathology, The First People’s Hospital of Xiaoshan District, Xiaoshan Affiliated Hospital of Wenzhou Medical University, Hangzhou, Zhejiang, China.

**Keywords:** computed tomography, highly differentiated follicular carcinoma, struma ovarii

## Abstract

**Rationale:**

Highly differentiated follicular carcinoma of ovarian origin (HDFCO) represents an exceptionally rare subtype of malignant struma ovarii. Despite its histologically indolent appearance, it retains metastatic potential. Early diagnosis, optimal management, and follow-up protocols can significantly reduce recurrence rates.

**Patient concerns:**

We report a 38-year-old female who was admitted to the hospital with lower abdominal pain for 1 month.

**Diagnoses:**

Preoperatively, the patient underwent pelvic ultrasound and computed tomography scans, which revealed multiple nodules and masses in the bilateral adnexal regions and abdominal cavity. The definitive histopathological diagnosis confirmed HDFCO.

**Interventions:**

The patient underwent total hysterectomy, bilateral adnexectomy, part of the greater omentum, pelvic mass excision and pelvic lymph node dissection, followed by total thyroidectomy and radioactive iodine therapy.

**Outcomes:**

The patient had an uneventful postoperative recovery. Serial measurements of serum thyroglobulin over the subsequent 3-year follow-up period consistently remained within the normal limits and showed no rising trend. Furthermore, all semiannual radiographic evaluations identified no signs of recurrence.

**Lessons:**

For the rare HDFCO, total thyroidectomy combined with radioactive iodine therapy should be considered following radical surgery to reduce the risk of recurrence. Meanwhile multidisciplinary collaboration is essential for the management of rare cases.

## 1. Introduction

Struma ovarii (SO) is a monodermal teratoma predominantly composed of thyroid tissue (>50%),^[1,2]^ accounting for approximately 2% to 5% of all ovarian teratomas.^[3]^ While the majority of SO cases are benign, malignant transformation occurs in about 5% to 10%, with papillary carcinoma being the most frequent subtype,^[4,5]^ followed by follicular carcinoma. Recently, a highly rare and distinct subtype – highly differentiated follicular carcinoma of ovarian origin (HDFCO) – has been described. HDFCO is characterized by the extraovarian dissemination of histologically benign-appearing thyroid follicular tissue. Despite its well-differentiated morphology, it is considered malignant due to its metastatic potential and can only be diagnosed upon the identification of extraovarian spread.^[6]^ However, the extreme rarity of HDFCO, combined with its nonspecific laboratory and imaging findings, poses significant diagnostic challenges. Furthermore, the absence of standardized treatment guidelines, attributable to its favorable prognosis and the limited number of reported cases, underscores the importance of documenting additional cases to inform clinical management. Herein, we report a case of a 38-year-old woman diagnosed with bilateral HDFCO and extensive abdominal dissemination, who was treated with thyroidectomy followed by radioactive iodine (RAI) therapy. We present a comprehensive analysis of the pathological characteristics, imaging findings, and clinical management of this rare HDFCO presentation.

## 2. Case presentation

A 38-year-old woman presented to our hospital with a 1-month history of persistent dull pain in her lower abdomen. She denied any nausea, vomiting, constipation, diarrhea, vaginal bleeding, or unintentional weight loss. Her history included 2 cesarean deliveries (2011, 2016) and laparoscopic ovarian cystectomy (2019, diagnosed as benign teratoma), recovering well after surgery. She is the mother of 2 healthy children and has no significant personal or family medical history, with both parents alive and in good health. Physical examination reveals a firm, fixed, fist-sized mass palpable in the right posterior aspect of the uterus, with bilateral adnexal thickening. Laboratory results showed elevated CA125 (51.30 kU/L; normal <35.0 kU/L), low thyroxine (45.45 nmol/L; normal 62.68–150.84 nmol/L), and low free thyroxine (8.59 pmol/L; normal 9.00–19.05 pmol/L). CEA, AFP, β-hCG, and LDH, TSH were normal.

**Figure 1.**
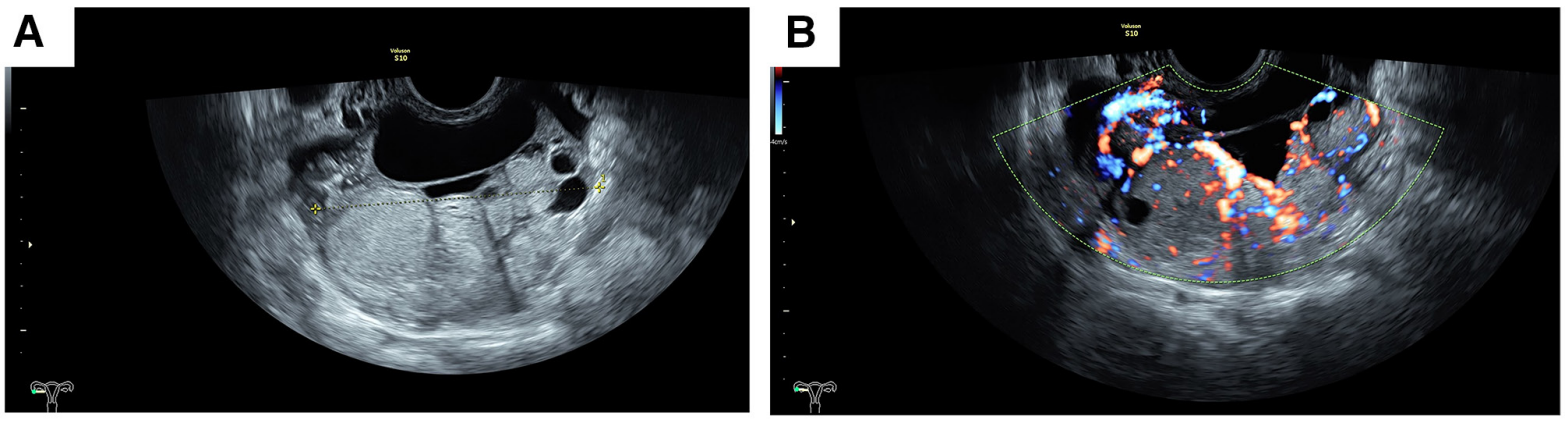
Pelvic ultrasound image. A solid mass in the right adnexal region (A), with abundant blood flow within it (B).

**Figure 2.**
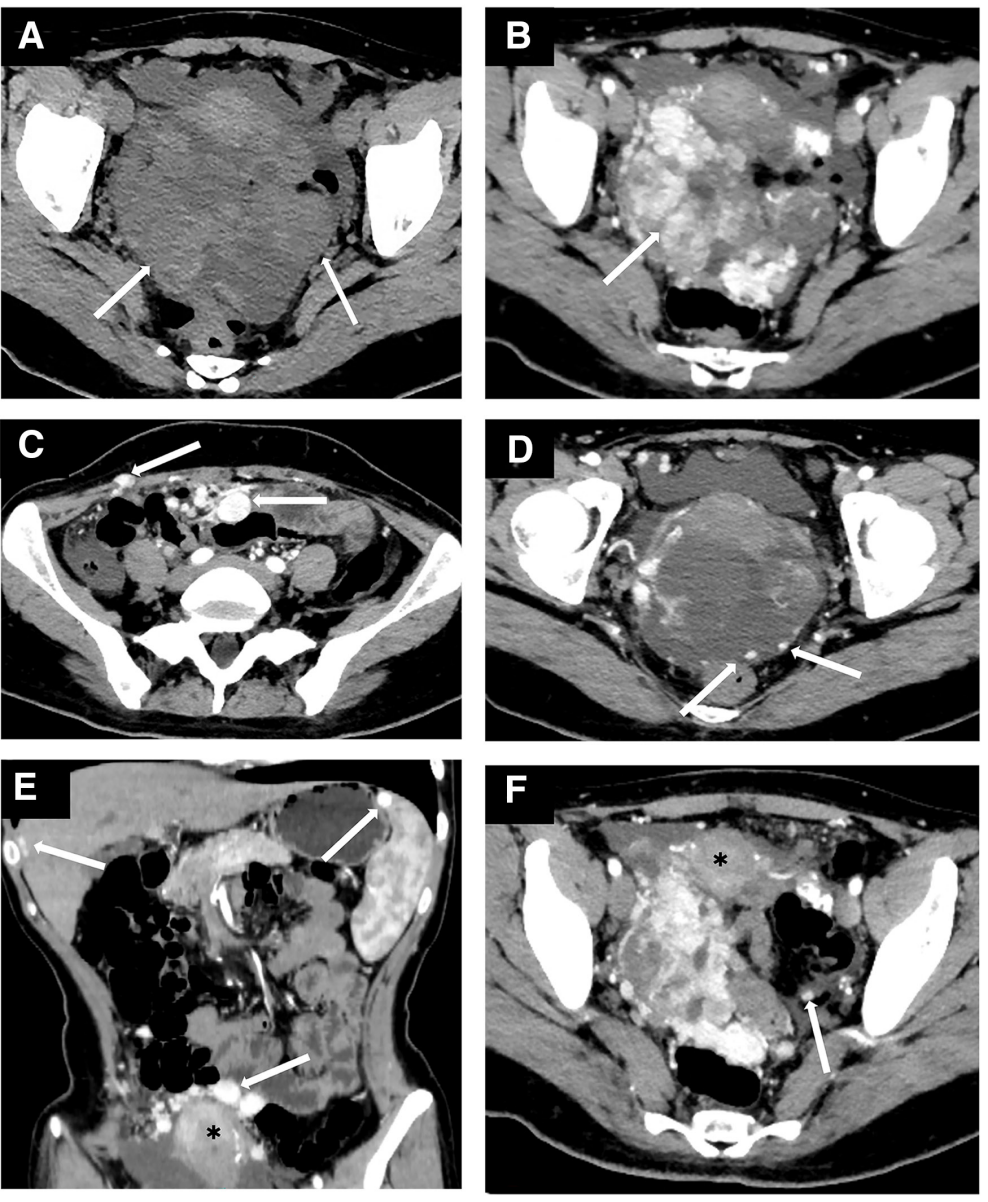
Abdominal CT image. (A) Non-contrast CT scan reveals bilateral adnexal masses. (B) Post-contrast imaging shows marked enhancement above the myometrium (black asterisk indicates uterus). Omentum (C), peritoneum (D), uterine serosal surface and hepatic/splenic capsule (E), sigmoid colon serosal surface (F) with multiple hyperenhancing nodules (white arrows indicate nodules). CT = computed tomography.

Pelvic ultrasound upon admission revealed cystic and solid masses in the bilateral adnexal regions. The largest cystic lesion (3.3 cm × 3.9 cm × 2.5 cm) and the largest solid mass (8.5 cm × 5.6 cm × 4.4 cm) were both located on the right side (Fig. 1A), with the solid component demonstrating rich vascularity on color doppler flow imaging (Fig. 1B). Unenhanced computed tomography (CT; Fig. 2A) reveals irregular, ill-defined complex cystic and solid masses in both adnexa, with cystic component attenuation of 10 to 28 HU (Hounsfield units) and solid component attenuation of 38 to 67 HU. Following contrast administration (Fig. 2B–D), the solid regions demonstrated marked and persistent enhancement that was significantly more intense than the myometrium. This “thyroid-like” enhancement pattern is a key imaging feature that helps distinguish SO from conventional epithelial ovarian cancer, which typically enhances less than the myometrium. Furthermore, the presence of extensive extra-ovarian disease strongly supported the diagnosis of malignant struma ovarii (MSO). Numerous intensely enhancing solid nodules were disseminated throughout the omentum, peritoneum, visceral serosal surfaces, and the anterior abdominal wall, with an enhancement pattern congruent with the primary ovarian masses. Notably, the absence of associated irregular peritoneal thickening helped differentiate this presentation from the more common peritoneal carcinomatosis of serous carcinoma. Pelvic ascites was also present. The CT findings were thus diagnostic of bilateral MSO with extensive abdominal metastasis. Thyroid ultrasound revealed no abnormalities.

The patient first underwent laparotomy under general anesthesia, and then partial resection of the left ovarian mass was performed, followed by intraoperative frozen section analysis. The definitive surgical procedure and extent will then be determined based on the tumor’s histopathological characteristics. The rapid frozen section pathology revealed well-differentiated adenoid structures of varying sizes with homogeneous cellular morphology, showing similarities to ovarian thyroid carcinoid tumors. Considering the malignant biological behavior of the patient’s ovarian tumor, following intraoperative discussions with the family, the surgical team performed a total hysterectomy, bilateral salpingo-oophorectomy, partial omental resection, pelvic mass excision, and pelvic lymphadenectomy. Intraoperative findings revealed a 7.0 cm × 6.0 cm × 5.0 cm cystic-solid mass arising from the right ovary, locally involving the intestinal tract and retroperitoneum. A 3.5 cm × 3.0 cm mass with characteristically friable texture was identified in the left ovary. Multiple masses of varying sizes were noted in the abdominopelvic cavity, with involvement of the sigmoid colon serosa, greater omentum, hepatic and splenic capsules, uterine serosa, and the right rectus abdominis muscle. Due to multiple intestinal involvement, the gastroenterology surgeon was consulted. It was considered that the mass was located on the surface of the rectum and there were no symptoms of intestinal obstruction at present, tumor resection could not achieve curative effect and was not removed temporarily. There was 200 mL of bloody ascites in the abdominal cavity. The surgery proceeded smoothly, and the patient recovered well from the surgery.

**Figure 3.**
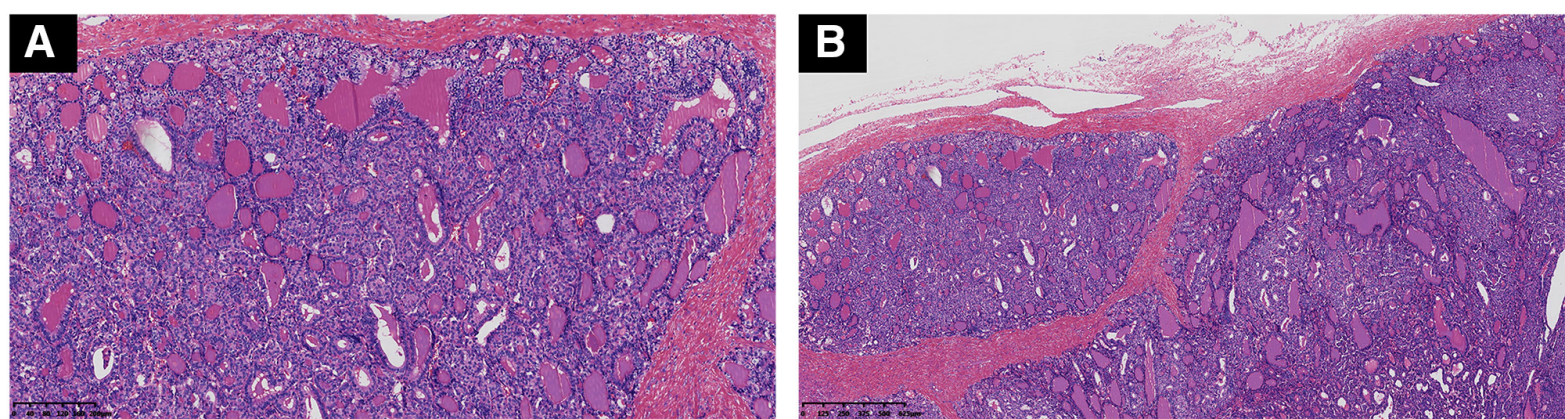
Pathological image. Thyroid follicular-like structures of varying sizes filled with colloid. (A) HE ×100, (B) HE ×40. HE = hematoxylin and eosin.

Postoperative histopathological (Fig. 3A, B): microscopic examination revealed well-differentiated follicular structures of varying sizes, composed of cells with uniform cytology. The morphological features bore a resemblance to those seen in a strumal carcinoid of the ovary. Immunohistochemical staining results: Positive for: Tg, TTF-1 (confirming thyroid origin); CD34 (indicating rich vascularity); CD56. Negative for: CgA, Syn, Inhibin-α (excluding medullary carcinoma and other endocrine tumors); HBME-1. The overall immunophenotype (CD56+/HBME-1-), combined with the histologically benign appearance of the extra-ovarian deposits, established the final pathological diagnosis of HDFCO.

Following confirmation of the diagnosis by the hospital’s multidisciplinary team (MDT), a treatment plan was formulated. Given the rarity of the disease and the absence of standardized postoperative guidelines for this FIGO stage IIIC highly differentiated follicular carcinoma, and considering its well-differentiated nature and presumed sensitivity to radioiodine, total thyroidectomy followed by RAI therapy was recommended. The patient subsequently underwent these procedures at an external institution (specific details unavailable). The patient has been closely monitored for a total of 3 years postoperatively. The initial follow-up at 2 months revealed normalized serum CA125 (8.50 kU/L) and thyroglobulin levels. Surveillance abdominal CT at that time demonstrated stable, enhancing paracolic nodules with no new lesions. The adjuvant RAI therapy was well-tolerated by the patient. She is maintained on a stringent surveillance protocol, which includes serial thyroglobulin measurements every 3 months and cross-sectional imaging every 6 months. All subsequent assessments to date have demonstrated sustained disease stability, with thyroglobulin levels within the normal range and no evidence of recurrence on the latest imaging.

## 3. Discussion

Struma ovarii originates from germ cells, and its pathogenesis may be related to the differentiation of germ cells into mature teratoma-type thyroid tissue, accounting for <1% of all ovarian tumors.^[2]^ Most SO cases are confirmed to be benign and only rarely undergo malignant transformation into MSO. This condition most frequently occurs in women aged 40 to 60 years and can, in some cases, be associated with clinical manifestations of hyperthyroidism. In this patient, preoperative thyroxine (T4) and free thyroxine (FT4) levels were both decreased, which may be associated with stimulation of the hypothalamic-pituitary-thyroid (HPT) axis by thyroid hormones secreted from the tumor tissue. Some cases are accompanied by ascites and elevated CA125 levels,^[3,7]^ a presentation fulfilling the criteria for pseudo-Meigs syndrome. This syndrome is likely caused by peritoneal irritation, which is also the presumed cause of the persistent abdominal distension and pain in our patient.

In the 5th edition (2020) of the World Health Organization (WHO) classification of female genital tumors, the term “HDFCO” was introduced. The pathogenesis of HDFCO remains poorly understood. However, hormonal fluctuations during pregnancy are considered a potential stimulus for thyrocyte proliferation.^[1,8]^ At the molecular level, Bao et al identified the FGFR4 Gly388Arg polymorphism as a possible significant genetic variant involved in tumorigenesis. Furthermore, the article describes a pathogenic continuum for HDFCO, conceptualized as a multistep malignant progression from an ordinary monodermal teratoma (OMCT) to SO and finally to HDFCO.^[8]^ This model offers a potential explanation for the malignant recurrence observed several years after the initial resection of a benign teratoma. The clinical course of our patient, who had a benign teratoma resected 3 years prior, aligns with this proposed spectrum. However, this association lacks robust validation. Roth et al confirmed that HDFCO exhibits indolent pathological features and lacks invasiveness, often remaining undetected until the tumor has spread beyond the ovary. The terminology used to describe this extra-ovarian involvement warrants discussion. Although the term “metastasis” is conventionally associated with aggressive, high-grade malignancies capable of distant dissemination, HDFCO demonstrates an indolent pathological profile and spreads primarily via direct extension and peritoneal implantation, typically lacking the destructive invasive growth observed in conventional carcinomas. Therefore, the term “spread,” as adopted in other key literature, may more accurately reflect its nonaggressive biological behavior and mode of dissemination. However, compared to previously reported cases, the tumor in our patient exhibited a notably more aggressive phenotype. This was characterized by its rare bilateral ovarian involvement and direct transperitoneal invasion into the abdominal wall musculature, a feature not previously documented in the available literature, to the best of our knowledge. Furthermore, the abdominal disease demonstrated extensive multiorgan involvement affecting the greater omentum, visceral serosa, and parietal peritoneum, substantially exceeding the scattered nodular distribution typically described in most existing series. Meanwhile, this report provides a detailed discussion on the imaging characteristics and differential diagnosis of MSO.

Struma ovarii typically presents as a multilocular cystic-solid mass with well-defined margins, smooth surface, and absence of mural nodules, the cyst walls often exhibit eggshell-like calcifications. The characteristic imaging feature is the “vacuum sign” - high-density cystic fluid on CT appearing as markedly hypointense signals on T2-weighted imaging (T2WI) sequences,^[9]^ reflecting the presence of viscous, colloid-rich material. The solid components interspersed within the cyst wall and septa, post-contrast enhancement demonstrates thyroid-like intense enhancement that is significantly stronger than myometrial enhancement, this reflects the rich vascularity of SO. The presence of extraovarian nodules or masses alongside SO is highly suggestive of MSO diagnosis. The differential diagnoses of MSO include ovarian carcinoma. While ovarian serous adenocarcinoma often presents with omental implants and elevated HE4 levels, typically manifesting as irregular cystic-solid masses or nodules with local invasion, peritoneal seeding, and occasional calcifications, but the degree of enhancement is significantly lower than that of MSO.

Due to the rarity of this disease, a consensus on its clinical management is lacking. Surgery remains the primary treatment, though debates persist regarding the optimal extent of resection and the management of thyroid tissue. The surgical approach spans a spectrum from conservative, uterus-preserving procedures to radical resections,^[1]^ with the choice heavily dependent on the clinical scenario, patient age, and fertility desires. For younger patients wishing to preserve fertility, fertility-sparing surgery is a viable option. In contrast, radical surgery – comprising total hysterectomy, bilateral salpingo-oophorectomy, resection of pelvic and omental masses, and para-aortic lymphadenectomy – is generally recommended for older patients or those with advanced-stage disease. Furthermore, minimally invasive surgery has been successfully applied in selected cases, underscoring its potential value.^[10]^ Postoperative management for most reported cases typically involves total thyroidectomy followed by RAI therapy.^[11]^ Kantreva et al documented 2 HDFCO patients who underwent this adjuvant regimen, both achieving favorable outcomes,^[3]^ which supports the rationale for combining thyroidectomy with RAI. Similarly, our patient received this combined therapy. The absence of recurrence in the residual abdominal lesions is likely attributable to the postoperative administration of I131. Although HDFCO generally carries a favorable prognosis, long-term recurrence risks necessitate extended follow-up exceeding 10 years, including serial thyroglobulin monitoring and abdominal imaging examinations.^[11]^

In summary, the management of HDFCO hinges on recognizing its malignant potential despite a deceptively benign histological appearance. For patients presenting with an ovarian tumor and peritoneal involvement, maintaining a high index of suspicion for HDFCO is essential, as this facilitates timely surgical intervention and necessary thyroid-specific therapy, including radioiodine treatment – measures critical to improving prognosis. Furthermore, a multidisciplinary collaborative approach plays a vital role in optimizing diagnosis and treatment.

## Author contributions

**Conceptualization:** Yonggang Qiu.

**Data curation:** Qiong Fu, Yonggang Qiu.

**Project administration:** Cuncheng Lou.

**Writing – original draft:** Lina Wang.

**Writing – review & editing:** Hao Dong, Yonggang Qiu.
